# Duodenal mucosal damage is associated with proliferative activity of Brunner’s gland hamartoma: a case report

**DOI:** 10.1186/1471-230X-14-14

**Published:** 2014-01-14

**Authors:** Mayumi Akaki, Shoji Taniguchi, Kinta Hatakeyama, Ryoji Kushima, Hiroaki Kataoka

**Affiliations:** 1 Clinical Laboratory, University of Miyazaki Hospital, 5200 Kihara, Kiyotake, Miyazaki, Miyazaki, 889-1692, Japan; 2 Section of Oncopathology and Regenerative Biology, Department of Pathology, Faculty of Medicine, University of Miyazaki, 5200 Kihara, Kiyotake, Miyazaki, Miyazaki, 889-1692, Japan; 3 Section of Surgery, Koga General Hospital, 1749-1 Sudaki, Ikeuchi, Miyazaki, Miyazaki, 880-0041, Japan; 4 Section of Pathophysiology, Department of Pathology, Faculty of Medicine, University of Miyazaki, 5200 Kihara, Kiyotake, Miyazaki, Miyazaki, 889-1692, Japan; 5 Pathology Division, National Cancer Center Hospital, 5-1-1, Tsukiji, Chuo-ku, Tokyo, 104-0045, Japan

**Keywords:** Brunner’s gland hamartoma, Brunner’s glands, Gastric foveolar metaplasia, MUC5AC, MIB-1, Mucosal damage

## Abstract

**Background:**

Brunner’s gland hamartoma is a rare tumor, predominantly found in the fifth to sixth decades of life. Generally, it is a single pedunculated polyp, rarely larger than 5 cm. Asymptomatic cases are found incidentally, but cases with a large polyp tend to have gastrointestinal bleeding and/or obstructive symptoms. Polyp size increases in a time-dependent manner, however, the growth mechanism is unknown. We report a Japanese male case in his mid-twenties with an over 6 cm sized polyp.

**Case presentation:**

A 26-year-old man presented black stools and anemia. Endoscopic examination revealed a large pedunculated polyp at gastroduodenal junction. The polyp, subsequently resected by distal gastrectomy, was lobulated with random surface erosions and sized 6.4 × 3 cm. Histological examination revealed that the polyp arose from duodenal mucosa and was composed of hyperplastic Brunner’s glands in lobules separated by fibromuscular septa, associated with lymphocytic infiltrate and lymphoid follicles. No evidence of malignancy was found. Thus, the lesion was diagnosed as Brunner’s gland hamartoma. Further immunohistochemical studies indicated that gastric foveolar metaplasia is associated with surface epithelium covering upper two thirds of the polyp, showing immunohistochemical positivity for mucin 5 AC (MUC5AC). Below the metaplastic surface epithelium, Brunner’s glands had high proliferative activity (MIB-1 labeling index: 7.9%). The similar staining pattern was observed at surface erosive sites (MIB-1 labeling index in Brunner’s glands: 9%). On the other hand, surface epithelium in the lower side of the polyp still preserved intestinal nature, containing CDX2-positive nuclei and MUC2-positive goblet cells. Brunner’s glands below the surface epithelium with intestinal characteristics showed low proliferative activity (MIB-1 labeling index: 0.77%).

**Conclusion:**

Proliferative activity of Brunner’s glands was high at the sites with surface erosion and also below the epithelium showing gastric foveolar metaplasia. As gastric foveolar metaplasia occurs along with a mucosal repair process in the duodenum, mucosal damages underlay the hamartomatous proliferation of Brunner’s glands and eventually resulted in a formation of large polypoid mass in this case.

## Background

Brunner’s gland hamartoma is a rare tumor-like lesion, predominantly seen in the fifth to sixth decades of life. This lesion is generally a single pedunculated polyp, with an average size of 2 cm, rarely larger than 5 cm, and locates in the first portion of duodenum [1]. Its pathogenesis is unknown and malignant change is rarely observed [2]. Most cases are asymptomatic and found incidentally. Cases with a large-sized polyp tend to have gastrointestinal bleeding and/or obstructive symptoms. According to studies with long-term follow-up, polyp size gradually increases in a time-dependent manner [3,4].

We experienced a 26 year-old male case with an over 6 cm sized polyp at gastroduodenal junction, investigated histological and immunohistochemical characteristics of the lesion, and report here.

## Case presentation

**Figure 1:**
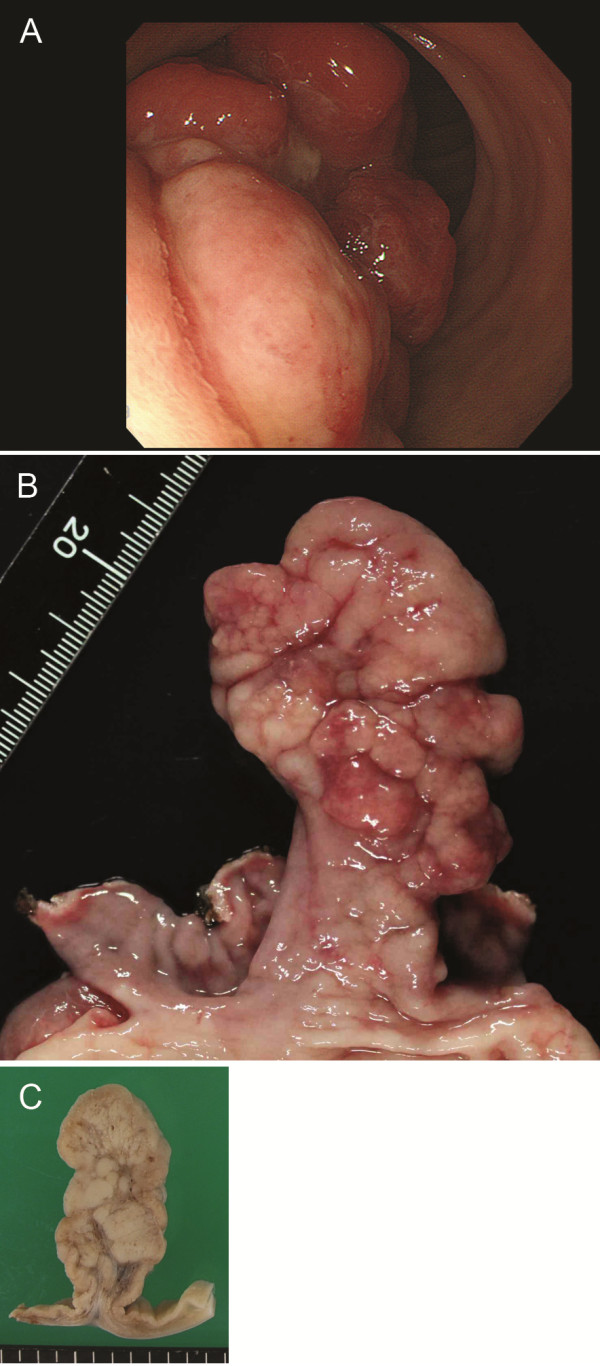
**Endoscopic image and gross findings. (A)** Endoscopic image showing a large polyp at gastroduodenal junction. The polyp head was incarcerated toward duodenal lumen. Hemorrhage was noted in part of the surface. **(B)** Surgically resected specimen showing a single lobulated and pedunculated polyp. Hemorrhage and erosion are associated. **(C)** Cut surface of the polyp is white and solid showing lobular structure and a broad fibrous stalk.

A 26-year-old man presented black stools and anemia. He had not taken any medication and had no specific family or past medical history. His height and weight were 172.3 cm and 93.4 kg (the body mass index was 31.5 kg/m^2^). His body temperature was 36.7°C, blood pressure was 137/77 mmHg and radial pulse rate was 80 beats/min and regular. Complete blood count showed the red blood cell count of 326 × 10^4^/μl, hemoglobin concentration of 8.6 g/dl (reference range: 13.5 to 17.6 g/dl), and mean corpuscular volume of 87 fl. Serum chemistry showed that hemoglobin A1c was 4.2% (National Glycohemoglobin Standardization Program number). Endoscopic examination of the upper digestive tract revealed a large pedunculated polyp arising from gastroduodenal junction, close to pyloric ring on the side between minor curvature and anterior wall (Figure 1A). The head of the polyp was incarcerated toward duodenal lumen. The lower side of the polyp showed hemorrhage in part. The stalk was too thick to be removed by endoscopic mucosal resection. Instead, distal gastrectomy with lymph node dissection was performed. No metastasis was found in the dissected lymph nodes.

The postoperative period was uneventful that the hemoglobin concentration gradually increased and became 11.6 g/dl on day 12. He was subsequently discharged without any complications and was in good health with 1 year follow-up.

**Figure 2:**
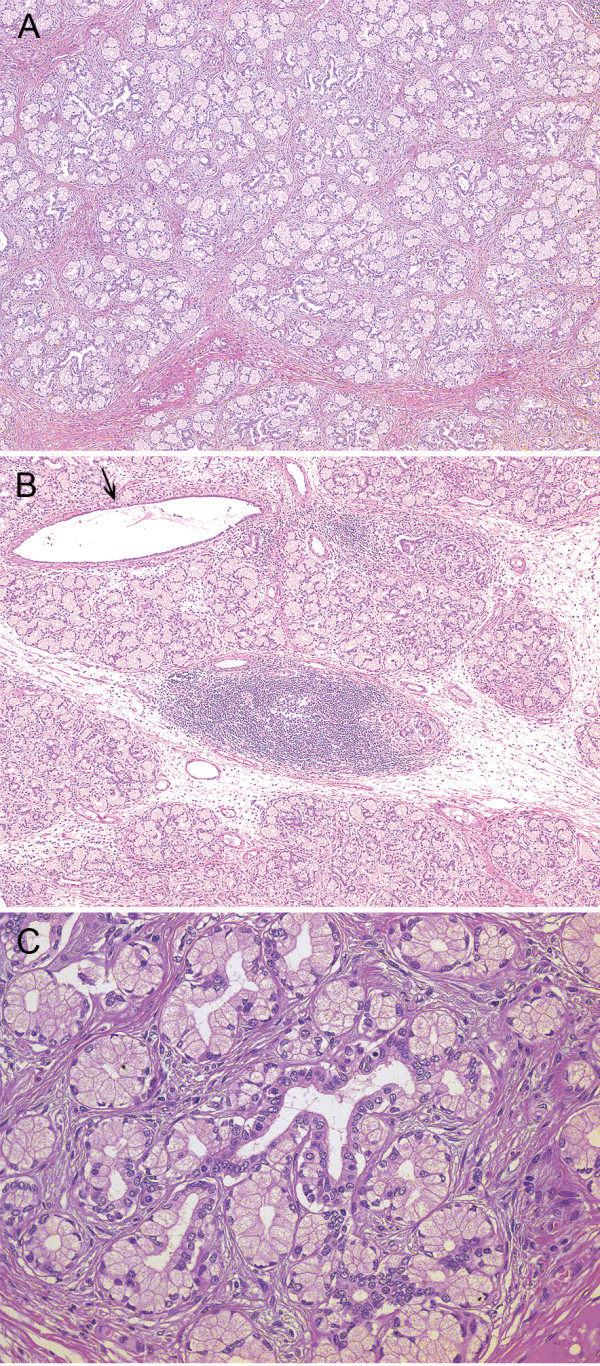
**Histological findings of the resected specimen. (A)** Hyperplastic Brunner’s glands in lobules are separated by fibromuscular septa. HE stain, 40× **(B)** Lymphocytic infiltrate is observed and a lymphoid follicle is formed between lobules of Brunner’s glands at the center. Arrow indicates a cystically dilated duct. HE stain, 40× **(C)** Acini and ducts are well-preserved without nuclear atypia. HE stain, 200×.

**Figure 3:**
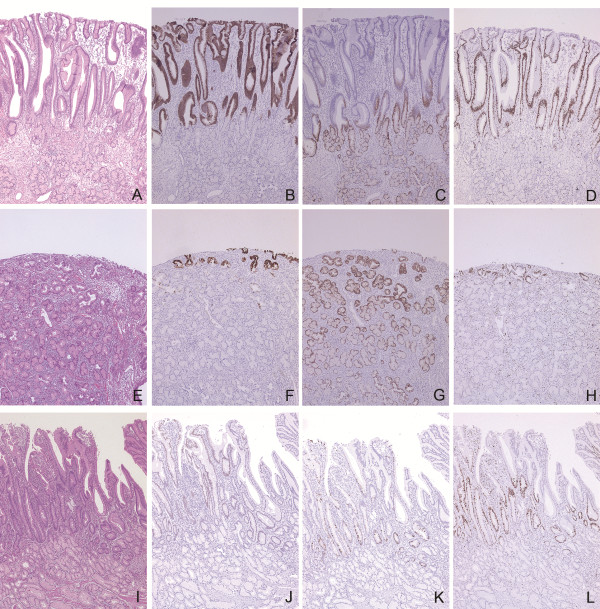
**Immunohistochemical findings of the resected specimen.** Duodenal mucosa in the upper part of the polyp **(A - D)**, with erosion **(E - H)**, and in the lower part of the polyp **(I - L)** are shown in HE stain **(A, E, and I)**, MUC5AC **(B and F)**, MUC6 **(C and G)**, MIB-1 **(D, H and L)**, CDX2 **(J)**, and MUC2 **(K)** immunostains. Surface epithelium in the upper part of the polyp is histologically similar to gastric foveolar epithelium and immunohistochemically positive for MUC5AC, suggesting gastric foveolar metaplasia **(B)**, and the epithelium in the deeper portion and Brunner’s glands are positive for MUC6 **(C)**. This area shows high MIB-1 labeling **(D)**. The similar staining pattern is observed in the mucosa with erosion **(E - H)**. Surface epithelium in the lower part of the polyp shows a villous structure, containing CDX2-positive nuclei **(J)** and MUC2-positive goblet cells **(K)**, suggesting preservation of the intestinal nature. MIB-1-positive Brunner’s glands are inconspicuous in this area **(L)**. All images are 40× magnification.

The surgically resected specimen showed a lobulated polyp sized 6.4 × 3 cm (Figure 1B) and the cut surface was solid and white (Figure 1C). Histologically, the stalk of the polyp projected from duodenal mucosa, not pyloric mucosa. In the lesion, marked proliferation of Brunner’s glands was noted showing lobular structures separated by fibromuscular septa (Figure 2A). Acini and ducts were well preserved (Figure 2C) and cystically dilated ducts were scattered (Figure 2B). Lymphocytic infiltrate was observed in the entire lesion and lymphoid follicle formation was intermingled in and between the lobules of hyperplastic Brunner’s glands (Figure 2B). The upper two thirds of the polyp was covered by a surface epithelium histologically similar to gastric foveolar epithelium (Figure 3A), randomly with surface erosions (Figure 3E). No evidence of malignancy was found within the specimen (Figure 2C). These findings suggested that the lesion was composed of normal tissue elements of duodenal mucosa and submucosa, but formed a disorganized aberrant mass. Thus, we diagnosed this lesion as Brunner’s gland hamartoma. Helicobacter pylori infection was not detected in Giemsa stain.

**Figure 4:**
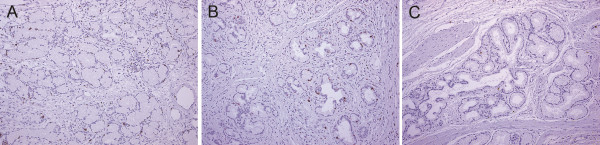
**MIB-1 labeling in Brunner’s glands.** Representative immunostain images of Brunner’s glands below surface epithelium associated with gastric foveolar metaplasia **(A)**, below erosion **(B)**, and below surface epithelium preserving intestinal nature **(C)** are shown. Brunner’s glands in **(C)** are rarely positive for MIB-1, compared to those in **(A)** and **(B)**. All images are 100× magnification.

Then, we sought to clarify the possible pathogenesis of this lesion by immunohistochemical analyses for mucin phenotypes, CDX2 expression and MIB-1 labeling. The surface epithelium resembling gastric foveolar epithelium was positive for MUC5AC (Figure 3B), indicating gastric foveolar metaplasia, while the epithelium in deeper portion and Brunner’s glands were positive for MUC6 (Figure 3C). The epithelial cells at the transition zone between MUC5AC- and MUC6-positive areas showed concomitant expression of MUC5AC and MUC6 (Figure 3B and C). These results confirmed that the surface epithelium differentiated towards gastric foveolar epithelium but preserved intestinal epithelial nature in the deeper portion. Interestingly, the MUC5AC-positive epithelium and adjacent MUC6-positive epithelium were diffusely positive for MIB-1 (Figure 3D), and the Brunner’s glands beneath them showed high MIB-1 labeling index (7.9%; Figures 3D and 4A).

The similar staining pattern was observed in the area with surface erosion (Figure 3E-H), where most of surface epithelium was lost, but the deeper portion of the epithelium was minimally left (Figure 3E), showing MUC5AC/MUC6 double positivity (Figure 3F and 3G) with significantly high MIB-1 labeling (Figure 3H). Moderately high MIB-1 labeling of Brunner’s glands (9%) was accompanied (Figures 3H and 4B).

On the other hand, surface epithelium in the lower part of the polyp showed a villous structure containing goblet cells without apparent gastric foveolar metaplasia (Figure 3I). Immunohistochemically, positivity of MUC5AC staining decreased in the lower one third of the polyp, particularly on the distal side. In the same area, CDX2-positive nuclei (Figure 3J) and MUC2-positive goblet cells (Figure 3K) were observed at the surface epithelium showing intestinal nature. Below the surface epithelium preserving intestinal nature, Brunner’s glands had less proliferative activity with low MIB-1 labeling index (0.77%) (Figures 3L and 4C). These results suggest that gastric foveolar differentiation is possibly related with the observed proliferative activity of Brunner’s glands.

## Conclusions

Brunner’s gland hamartoma is generally a single pedunculated polyp composed of hyperplastic Brunner’s glands with a variable amount of ducts, smooth muscles, fibrous tissue, adipose tissue, and lymphocytes. This lesion is interchangeably called as Brunner’s gland adenoma, however, some dispute using the latter because this lesion is usually an admixture of normal tissues without cytological atypia.

Brunner’s gland hamartoma is predominantly found in those in their fifties to sixties. Several cases in their twenties have been reported with or without describing the lesion size, and to the best of our knowledge, only two cases (23- and 20-year-old women) have been reported mentioning their unusually large polyp size, 5.5 cm examined histologically and 7 cm observed by endoscopy [5,6].

The major disadvantage having a large polyp is that a benign lesion may be misdiagnosed and over-treated as malignancy. In the past, a 60-year-old male case was reported that Brunner’s gland hyperplasia forming a 5.5 cm polyp at duodenal bulb was treated by duodenocephalopancreatectomy [7]. When a large polyp is noted at duodenum, it is important for clinicians to have Brunner’s gland hamartoma as a differential diagnosis, even with the rarity of the disease.

In our immunohistochemical study, proliferative activity of Brunner’s glands was high beneath the epithelium showing gastric foveolar metaplasia. Gastric foveolar metaplasia seems to be a common finding in Brunner’s gland hamartoma [2], and also associated with dysplastic changes in Brunner’s gland hyperplasia [8,9]. One of the duodenal diseases frequently with gastric foveolar metaplasia is duodenal ulcer. In a duodenal ulcer lesion, mucosal repair mechanism initially generates epithelium with gastric foveolar metaplasia and then expands Brunner’s glands [10]. Although how Brunner’s gland hamartoma grows is unclear, it is reasonable to assume that repeated mucosal damages activate mucosal repair mechanism, facilitating a proliferation of Brunner’s glands accompanied by surface gastric foveolar metaplasia.

Kim, *et al.*, recently reported histology and mucin expression in Brunner’s gland hamartoma and hyperplasia [2]. They showed that Brunner’s gland hamartoma/hyperplasia lesions tended to be larger with surface epithelial change, such as erosion, ulcer, and gastric foveolar metaplasia, which is in accordance with our hypothesis that mucosal damage facilitates Brunner’s gland proliferation. Although proliferative activity of Brunner’s glands near surface epithelium was not described in their study, slightly increased proliferative activities in atypical glands and sclerotic glandular foci were reported with MIB-1 labeling index of 3% and 2%, respectively. The sclerotic glandular foci are probably old lesions in the mucosal repair process, since the foci were found in 63.2% of the cases with surface epithelial changes but not in the cases without surface epithelial change [2].

Considering possible causes of mucosal damage, mechanical stimuli, Helicobacter pylori infection, and hyperacidic environment in duodenum have been suggested. The latter two have been postulated to be involved in the development of Brunner’s gland hamartoma [11,12]. Helicobacter pylori infection was reported in some cases of Brunner’s gland hamartoma [3], but not observed in the other cases [4]. The infection is unlikely to be essential for a hamartomatous proliferation of Brunner’s glands. Hyperacidity can induce hyperplastic change of Brunner’s glands, which secrete alkaline mucin to buffer gastric acid. Franzin, *et al*., demonstrated hyperacidic state in patients with Brunner’s gland hamartoma [12]. Interestingly, gastric foveolar metaplasia has been regarded as a protective change of the duodenal mucosa against hyperacidity [13].

In summary, we report a case of large Brunner’s gland hamartoma in a young (26-year-old) patient. Our immunohistochemical results suggest that mucosal damage underlies the hamartomatous proliferation of Brunner’s glands.

## Consent

Written informed consent was obtained from the patient for publication of this case report and any accompanying images. A copy of the written consent is available for review by the Series Editor of this journal.

## Abbreviations

MUC: Mucin; HE: Hematoxylin and eosin.

## Competing interests

The authors declare that they have no competing interests.

## Authors’ contributions

MA collected and analyzed the data, and was involved in drafting the manuscript. ST did clinical aspects of this study including surgical treatment. KH helped designing this study. RK guided us for pathological diagnosis and conception. HK revised the manuscript critically for important intellectual content and supported financially. All authors read and approved of the final manuscript.

## Pre-publication history

The pre-publication history for this paper can be accessed here:

http://www.biomedcentral.com/1471-230X/14/14/prepub
